# Maternal Anaemia at Delivery and Haemoglobin Evolution in Children during Their First 18 Months of Life Using Latent Class Analysis

**DOI:** 10.1371/journal.pone.0050136

**Published:** 2012-11-21

**Authors:** Kobto G. Koura, Smaïla Ouédraogo, Gilles Cottrell, Agnès Le Port, Achille Massougbodji, André Garcia

**Affiliations:** 1 IRD UMR216, Mère et enfant face aux infections tropicales, Paris, France; 2 Faculté de Pharmacie, Université Paris Descartes, Paris, France; 3 Université Pierre Marie Curie, Paris, France; 4 Faculté des Sciences de la Santé, Université d’Abomey-Calavi, Cotonou, Bénin; 5 Centre d’Etudes et de Recherche sur le Paludisme Associé à la Grossesse et à l’Enfant, Cotonou, Bénin; University of Massachusetts Medical School, United States of America

## Abstract

**Background:**

Anaemia during pregnancy and at delivery is an important public health problem in low- and middle-income countries. Its association with the children’s haemoglobin level over time remains unclear. Our goals were to identify distinct haemoglobin level trajectories using latent class analysis and to assess the association between these trajectories and maternal anaemia and other risk factors.

**Method:**

A prospective study of children from birth to 18 months of life was conducted in a rural setting in Tori-Bossito, Benin. The main outcome measure was the haemoglobin levels repeatedly measured at 3, 6, 9, 12, 15 and 18 months. Variables were collected from the mothers at delivery and from their children at birth and during the follow-up. The analyses were performed by means of Latent Class Analysis which has never been used for this kind of data. All the analyses were performed with Stata software, version 11.0, using the generalized linear latent and mixed model (GLLAMM) framework.

**Results:**

We showed that 33.7% of children followed a low haemoglobin trajectory and 66.3% a high trajectory during the first 18 months of life. Newborn anaemia, placental malaria, malaria attack, sickle cell trait and male gender were significantly associated with a lower children’s haemoglobin level over time, whereas maternal age, children living in a polygamous family and with good feeding practices had a higher Hb level in the first18 months. We also showed that maternal anaemia was a predictor for ‘low haemoglobin level trajectory’ group membership but have no significant effect on children haemoglobin level over time.

**Conclusion:**

Latent Class Analyses framework seems well suited to analyse longitudinal data under the hypothesis that different subpopulations of subjects are present in the data, each with its own set of parameters, with distinctive evolutions that themselves may reflect distinctive aetiologies.

## Introduction

Anaemia during pregnancy and at delivery, defined by haemoglobin (Hb) concentration lower than 11 g/dl, is an important public health problem in low-income and middle-income countries [1]. In 2009, McLean *et al. e*stimated that 30–60% of pregnant women were anaemic [2].

The consequences of maternal anaemia on pregnancy outcomes are well documented despite the existence of discrepant results. Studies have shown a relation between maternal anaemia during pregnancy and low birth weight, preterm birth and newborn anaemia [3]–[5]. However, its association with the children’s haemoglobin level over time remains unclear. In 2002, de Pee *et al.* showed a relation between the mother’s haemoglobin concentration during pregnancy and the infant’s haemoglobin level from 3 to 5 months of age [6]. Moreover, iron-deficiency anaemia was also found to be more frequent in children born to anaemic mothers than children born to non-anaemic mothers in Jordan, Indonesia and Niger [6]–[8]. All previous studies used cross-sectional analyses not taking into account the correlation between Hb measurements taken repeatedly on the same individuals.

Between 2007 and 2010, more than 2500 Hb measurements were collected from 542 Beninese children enrolled in the Tori Bossito project and followed-up from birth to 18 months [9], [10]. Our objective in this study was to examine factors that can influence children haemoglobin level with special emphasis on the effect of maternal anaemia at delivery. During a first analysis of these data, using hierarchical mixed models to deal with the repeated measures design, we concluded that the evolution of haemoglobin levels from birth to 18 months of age was associated with several children’s characteristics but not with maternal anaemia at delivery [11].

However, mixed models, although well suited to longitudinal data analysis, make the important assumption that the individuals in the sample are randomly drawn from a homogeneous sample (with an allowed individual variability accounted by the covariates and the random effects). Nevertheless, in this sample, in which more than 60% of newborns were anaemic at birth and a large proportion remained anaemic during the follow-up, it seemed to us very interesting to test whether distinct subpopulations of children are present in the data, each with its own set of parameters, with distinctive haemoglobin evolutions, or trajectories, that themselves may reflect distinctive aetiologies. The identification of trajectories of haemoglobin through the first 18 months of life and risk factors that predispose to or modify a particular trajectory may provide a better understanding of the natural history of how children’s haemoglobin evolves. There are several ways to model mixture of populations and latent class analysis (LCA) approach is one of them. However, all these approaches allow exploring this issue partly because they do not assume that all individuals belong to the same homogeneous population. It allows us to identify latent groups of individuals who share a particular developmental trajectory of some attribute. [12], [13].

Thus, the main objective of the present work was to provide a new approach to analyze the existence and characteristics of groups of children with similar patterns of Hb levels evolution between 3 and 18 months of life in a cohort of Beninese children included in the Tori Bossito project.

## Methods

### Study Design

The Tori Bossito project is a birth cohort study of children who were followed up in the first18 months of life. Full details of the survey have been described elsewhere [9]–[11].

### Study Site and Population

This study was conducted in nine villages in the district of Tori-Bossito, a semi-rural area located 50 km north of Cotonou, the economic capital of Benin. The study participants were recruited in three health centres (Tori Avame, Tori Cada and Tori Gare) in the districts, which were chosen because of their capacity to provide adequate care to children and their proximity to the study population’s residential area. Malaria is perennial and *Plasmodium falciparum* is the commonest species. There are two high transmission peaks from April to July and October to November. Transmission is low the rest of the year.

**Figure 1:**
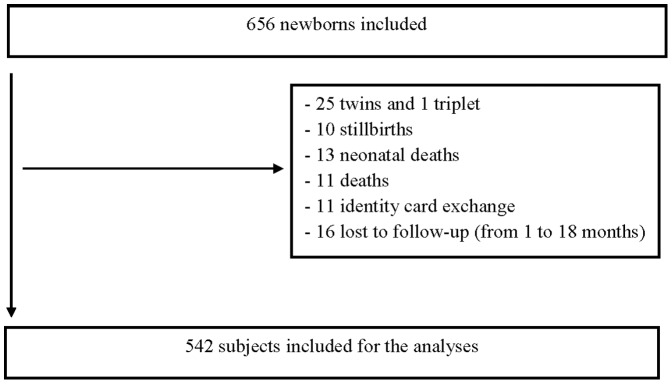
Enrollment and follow-up in study, Tori-Bossito, Benin, 2007–2010.

The study population was composed of pregnant women who came to any of the three health centres for delivery between June 2007 and July 2008. The newborns included were followed up until January 2010 (Figure 1).

### Variables Measured

#### Variable of interest: Hb

At 3, 6, 9, 12, 15 and 18 months of life, the haemoglobin level was assessed on a por device (HemoCue®) using a drop of blood. The haemoglobin level repeatedly measured at 3, 6, 9, 12, 15 and 18 months was our outcome variable.

#### Covariates

The following variables were collected from the mothers at delivery: (1) maternal anaemia at delivery defined as Hb level less than 11 g/dl (Hb level was measured before birth ), (2) age, (3) ethnic group, (4) working status during pregnancy (housewife vs. working), (5) maternal marital status (monogamous vs. polygamous family), (6) parity (primipare vs. multipare), (7) number of prenatal care visits (less or more than four prenatal visits), (8) educational status (schooled vs. unschooled) and (9) placental malaria (thick and thin placental smears were made to look for placental malaria). The following variables were collected from the children: (1) newborn’s anaemia (Hb<14 g/dl), (2) low birth weight (<2500 g), (3) preterm birth (gestational age<37 completed weeks of gestation), (4) gender, (5) sickle cell trait, (6) number of malaria attacks during the follow-up and (7) nutritional status of children during all the following-up period, using a WHO/UNICEF indicator [14] : infant and young child feeding indicator (IYCF) is the sum of minimum meal frequency and minimum dietary diversity. For each child and each month, based on dietary recall of the last 24 hours we calculated the nutrition score which was equal to 1 when the IYCF requirement was satisfied.

### Statistical Analysis

We used latent class analysis to identify the haemoglobin level trajectories and assess their association with the covariates.

A general model’s formulation is:
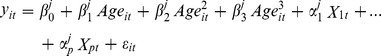
where 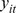 is the response variable (Hb level of the 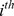 children at age *t*), the 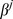 are the coefficient associated to the children’s age in the 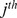 group and the 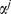 are the coefficient associated to covariates in the 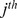 group (the index *t* specifies that the covariates can depend on time) and 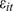 the residual variation [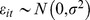].

The posterior probability 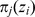 that an children 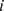 with the covariates vector 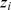 belongs to the group 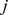 is: 
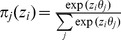
 with covariates vector 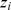 and its corresponding coefficients vector 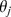.

The analysis was performed in four steps.

In the first step, each haemoglobin level trajectory was modelled using a polynomial function of time and no covariates were added to the model at this time. The most appropriate number of classes was determined using the Bayesian Information Criterion (BIC). The substantive criteria included the group prevalence greater than 5% of the sample because of the usefulness and practical interpretability of latent classes [15].

In the second step, we explored the relationships between haemoglobin level trajectories and the covariates. All covariates that were significantly associated with haemoglobin level in univariate analysis with *p*<0.20 were entered in the multivariate analysis. Statistical significance in the final model was set at *p*<0.05. During this step, we examined the stability of the latent classes determined in the first step. Indeed, as mentioned by Nagin the percentage of subjects in each trajectory must not widely change when the covariates are successively added to the model [16]. In addition we apply the same substantive criteria as previously [15]. Moreover, to check if the introduction of a further group yields improves the analysis, we used the Gateaux derivative method allowing testing whether the introduction of a new group increases the likelihood when all other parameters are held constant at their previous maximum likelihood values [17], [18]. In the final model, after multivariate analysis, each individual was assigned to the trajectory group for which he had the highest posterior probability. Within each group, posterior probability values greater than or equal to 0.7 indicate adequate internal reliability [15]. Taking together, these criteria allowed us to make a definitive choice of the appropriate number of classes.

In the third step, we studied the predictor effect of maternal anaemia to belong to one or more trajectory depending on the number of classes we identified.

Finally, our fourth step consisted to test whether the effects of covariates on the evolution of haemoglobin levels in the first18 months of age differed within each trajectory.

All these analyses were performed with Stata software, version 11.0 (StatCorp LP, College Station, TX, USA), using the generalized linear latent and mixed model (GLLAMM) framework [19], [20].

### Ethics

The study’s protocol was approved by the University of Abomey-Calavi’s institutional review board and the IRD’s Consultative Ethics Committee. All women who participated in this study signed informed consent before enrolment (which also included their children) and were able to withdraw their consent at any time during the study.

## Results

The analysis covered 2708 haemoglobin measurements out of the expected 3252. In most cases, an Hb measurement was missing from the children’s follow-up because the mother had not attended a quarterly meeting: 480 children (89%) were present at least four times out of six at quarterly meetings during the follow-up. Full details of the baseline characteristics of the subjects have been described elsewhere [11].

First we identified groups of individuals who followed a similar pattern of haemoglobin from age 3 to 18 months. Using the BIC as criteria, the model with three groups was selected when compared to the two-group model (8289.1 and 8345.1, respectively). A fourth group, when added, accounted for less than 0.6% of the children. The same pattern was observed with models of more than four groups (i.e. less than 1% of the sample fell into the supplementary trajectories). In the two-group model, the first latent trajectory accounted for 32.8% of children with a low haemoglobin level and the second trajectory 67.2% of children falling into the highest haemoglobin level in the first18 months of life. For the three-group model, the first latent trajectory accounted for 10.6% of the children with the lowest haemoglobin level. The second latent trajectory accounted for 34.3% of the sample falling into an intermediate haemoglobin level category and, finally, the third latent trajectory accounted for 55.1% of the children who fell into the highest haemoglobin class.

**Figure 2:**
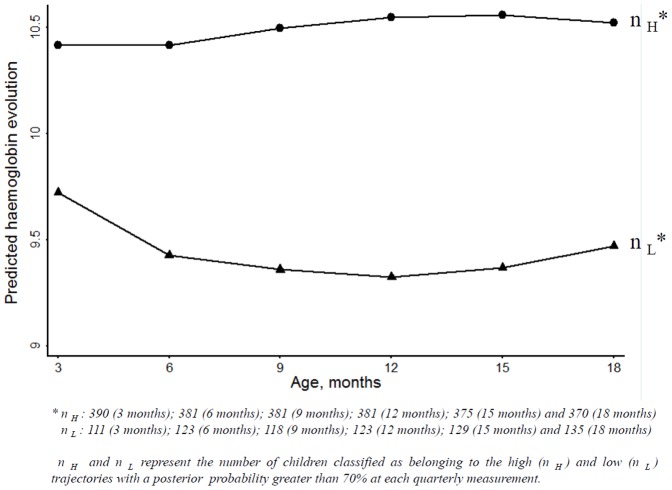
Trajectories for haemoglobin (3 to 18 months of age).

Then we explored the relationships between haemoglobin trajectories and covariates using the two-group model and the three-group model. The effects of covariates were similar with the two- or three-trajectory models (see below). However, after adding covariates in the three-trajectory model, only 2% of the sample fell into the lowest haemoglobin trajectory instead of 10.6% previously, consistent with the instability of the three-trajectory model. The Gateaux derivative method confirmed that the three-point solution may represent a local maximum of the log-likelihood. Finally, for the following analyses, we used the two-group model. The largest group identified, called the ‘high trajectory’ group, accounted for 66.3% of the children in this sample. The second trajectory included children who had lower haemoglobin from 3 to 18 months of life. This group was called the ‘low trajectory’ and accounted for 33.7% of the sample. With this model, 95.2% of children from the high haemoglobin level trajectory were classified as belonging to this group with a posterior probability greater than 70%, whereas 86.0% of children from the low haemoglobin level trajectory were classified as belonging to this group with a posterior probability greater than 70%. These two groups are presented in Figure 2 and were labelled according to their most unique characteristics. For the low trajectory, the haemoglobin level at 3 months of age was around 9.75 g/dl and decreased between 3 and 12 months; After 12 months it increased slightly until 18 months. For the high trajectory, the haemoglobin level started around 10.4 g/dl and increased from 3 to 12 months of life and remains stable for the last six months. Table 1 showed the descriptive variables of these two latent groups.The factors associated with children haemoglobin progression in multivariate analysis are presented in Table 2. Newborn anaemia, placental malaria, malaria attack, sickle cell trait and male gender were significantly associated with a lower haemoglobin level in the first18 months of age (*p* = 0.007, *p* = 0.039, *p* = 0.005, *p*<10^−3^ and *p = *0.012, respectively). Maternal age, children living in a polygamous family and with good feeding practices had a higher Hb level in the first18 months of age than others (*p = *0.013, *p* = 0.01 and *p* = 0.003, respectively). We found that maternal anaemia at delivery had no significant effect on children haemoglobin level in the first18 months (*p* = 0.275).

**Table 1:** Table of descriptive variables of the two latent groups.

	High trajectory	Low trajectory
Birth weight, mean (SD)	2994.8 (382.8)	2951.6 (384.2)
Low Birth weight	32 (7.8%)	18 (13.6%)
Preterm birth	43 (10.5%)	12 (9.2%)
Hemoglobin rate, mean (SD)	14.6 (1.9)	14.2 (1.9)
Newborn’s anemia	143 (35.5%)	49 (37.7%)
Children’s gender (Female)	191 (46.8%)	81 (61.8%)
Sickle cell trait (carrier or not of AS variant)	80 (20.1%)	32 (26.0%)
Number of malaria attacks during the first 18 months		
0	147 (36%)	36 (27.5%)
1 to 2	176 (43%)	61 (46.5%)
>2	85 (21%)	34 (26%)

**Table 2:** Risk factors for children’s haemoglobin progression from 3 to 18 months of life in each latent class identified by the Latent Class Analysis, Benin, 2007–2010.

Covariates	Estimation	StandardErrors	*p*-value
Intercept	10.17	0.11	<10^−3^
Maternal anaemia			
(Reference = No)			
Yes	−0.07	0.06	0.275
Newborn anaemia			
(Reference = No)			
Yes	−0.18	0.06	0.007
Placental malaria			
(Reference = No)			
Yes	−0.20	0.09	0.039
Number of children’s malaria attack			
(Reference = 0)			
1 to 2	−0.15	0.07	
>2	−0.26	0.08	0.005
Children’s gender			
(Reference = Female)			
Male	−0.15	0.06	0.012
Maternal marital status			
(Reference = Monogamous family)			
Polygamous family	0.17	0.07	0.01
Sickle cell trait			
(Reference = No)			
Yes	−0.27	0.07	<10^−3^
Maternal age			
(Reference = Age ≤20)			
21–25	0.25	0.10	
26–30	0.28	0.10	
≥30	0.26	0.10	0.013
Children's feeding in the first18 months	0.17	0.06	0.003

In the third step of our analysis, we examined the role of maternal anaemia as predictor of belonging preferentially to either group. Children born to mothers with maternal anaemia were more likely to belong to the lower trajectory class [OR = 1.65, *p* = 0.03].

Finally, among the covariates significantly associated with children haemoglobin in the first18 months, sickle cell trait was the only one for which the effect differed within each group (Table 3). Indeed, its negative effect was more strongly marked within the low trajectory (*p* = 0.023).

**Table 3:** Effects of covariates among trajectories.

Variable	High trajectory	Low trajectory	*p-value* *
Newborn anaemia	−0.02	0.04	0.589
Placental malaria	−0.0003	0.08	0.543
Number of children’s malaria attack	−0.0248	0.001	0.988
Children’s gender	−0.003	0.05	0.458
Maternal marital status	0.12	0.07	0.36
Sickle cell trait	−0.037	−0.25	0.023
Maternal age	−0.046	0.08	0.25
Children's feeding in the first18 months	0.087	0.095	0.326

* The null hypothesis is that the covariate has the same effect for each trajectory. A significant p-value is consistent with a different effect of the covariate for each group.

When maternal anaemia at delivery and newborn anaemia were replaced respectively by haemoglobin levels as quantitative variables, the same pattern of results was obtained. Maternal haemoglobin level at delivery was not significantly associated with children haemoglobin level in the first18 months (*p* = 0.8) whereas newborn haemoglobin level had significant effect (*p* = 0.03).

## Discussion

As far as we know, published data describing repeated haemoglobin measurements from children over extended periods are very scarce. Only one analytic method for such data has been proposed heretofore [11], [21]. We previously showed that neither haemoglobin level at birth nor maternal anaemia were associated with children Hb level in the first18 months, but the occurrence of a malaria attack during follow-up, male gender and sickle cell trait carriage were associated with a lower children haemoglobin level in the first18 months. Children living in a polygamous family and with good feeding practices had a higher Hb level in the first18 months of age than the others. The present analysis, using latent class models, confirms these results, strengthening the importance of these risk factors, which have been discussed elsewhere [11]. However, this approach, one among several population mixture models, provided a better understanding of the natural history of children haemoglobin levels by identifying new determinants of haemoglobin levels (i.e. placental malaria and the newborn anaemia) as well as the role played by mother’s anaemia as a predictor of belonging to the low latent trajectory. We also showed that the negative effect of sickle cell trait was more strongly marked within the low trajectory. In our preceding analysis using mixed models, we found that newborn anaemia and placental malaria were associated with a decreased haemoglobin level in the first 18 months of life. However, these associations were not significant (*p* = 0.104 and *p* = 0.190 respectively). This result strengthens the fact that taking into account the existence of a mixture of populations could help to identify covariates with significant but probably complex effect that cannot be easily identified under the hypothesis that all subjects belong to the same population. Finally, the innovative LCA suggests that the data are compatible with two levels of haemoglobin over time, high and low, concerning two-thirds and one-third of the sample, respectively.

Latent class analysis has been used extensively in criminology and behavioural research [22]–[24], less to date in public health research [25]–[27]. It is an extension of a mixed model and assumes the presence of and identifies latent groups of individuals who share a particular developmental trajectory of some attribute, thereby allowing a better understanding of the pattern of change in the variable of interest [15], [16], [28]–[30]. The other strength of this approach is that it reveals a predictor of belonging to the low haemoglobin group along with the effects of all other risk factors. Furthermore, it allows studying the effect of variables within trajectories and underlined the negative influence of sickle cell trait during infancy. Latent class analysis forms a part of mixture modeling, a widely applied data analysis approach used to identify unobserved heterogeneity in a population. Mixture modeling involved several techniques with potential differences [31]. We provided our model equation to take these potential differences into account and to make our results easily and clearly understood, despite technical considerations, with no risk of miss understanding. One limitation of our approach could be that each group has the same variance structure rather than a variance-covariance structure among time points.

Multiple indices have been described to identify clusters in Latent Class Analysis. To date, there is not common acceptance of the best criteria for determining the number of classes in mixture modeling, despite various suggestions [32]. Among them, several simulation studies indicated that the BIC performs well [33], [34]. More recently, Nylund et al. found that the Bootstrap Likelihood ratio test (BLRT) presented the best performances and that the second best index was the BIC. They also showed that the BLRT, does have its disadvantages such as the increased computation time [32], but this should not affect the fact that it is more powerful and accurate. Finally, the authors strongly recommended using the BIC as the first step. Here, we followed this recommendation, together with the criteria proposed by Andruff [15] based on the percentage of population included in each group. However, despite the fact that they are widely used in the literature [22], [27], [35], as they are partly based on the percentage of the sample within the trajectories, this choice is somewhat subjective. To take this point into account, we also used the Gateaux derivative method to determine the number of groups required to achieve the largest possible likelihood. This method has confirmed our choice of two trajectories for the changes in haemoglobin level, suggesting that the three-point solution may represent a local maximum of the log-likelihood.

Finally, to take into account the complexity and the possibilities of these population mixture models, it could be of great interest to pursue this exploration by using more indices to define the number of clusters and by adding a variance-covariance structure among time points. This could be proposed by using and comparing several modelling mixture methods in a near future for such complex data.

In the present study, maternal anaemia at delivery seems to be predictive of belonging to the low trajectory but has no direct effect on the changes in haemoglobin level over time. The association we found between the effect of maternal anaemia at delivery, and the absence of direct effect on the evolution of haemoglobin level during infancy, could apparently seems contradictory. Indeed this trajectory loses Hb over time, whereas the higher trajectory does not. One possible explanation could be that the association between maternal anaemia at delivery and haemoglobin level during infancy could be (at least partly) mediated through newborn anaemia. Indeed, inserting both maternal anaemia and newborn anaemia in the same model could result in collinearity, which could explain the apparent absence of effect of maternal anaemia on children haemoglobin evolution. However, maternal anaemia remained not significantly associated (*p* = 0.14) with the evolution of children haemoglobin progression even when included in a multivariate model without newborn anaemia. Furthermore, using the Bayesian Informative Criterion as criteria, the model with newborn anaemia was selected when compared to the model with maternal anaemia. Using haemoglobin level of mother at delivery as quantitative variable did not change these results (data not shown). Hence, this pattern of results can be interpreted as the fact that maternal anaemia stops having a negative effect at birth but that children born of an anaemic mother are probably disadvantaged during infancy. The mother’s anaemia could also be interpreted as an indirect marker of a woman’s and/or a family’s disadvantage because of poor socioeconomic status that could be associated with inadequate nutrition during infancy. The importance of adequate nutrition is illustrated by the positive effect of nutritional status on the haemoglobin progression during infancy.

The mechanisms by which a newborn’s anaemia at birth can affect haemoglobin level over time have not yet been clearly described. A period of rapid growth, especially during infancy, results in substantial demands for iron. In developed countries where breastfeeding is not common, mothers often use children formula fortified with iron in order to supplement the children’s needs. This is not necessary with breastfeeding, which provides high concentrations of highly absorbable iron. In developing countries where all children are routinely breastfed during the first year of life, we could expect that breastfeeding compensates iron requirements and corrects anaemia. However, the interactions between iron intakes and stores are complex and it has been shown that exclusive breastfeeding at 4 months of life was protective of iron status and of iron deficiency-anaemia at 6 months, compared with children receiving early complementary food [36]. In our study population, breastfeeding is far from exclusive, and at 4 months of age only 18% of children were exclusively breastfed (data not shown). Moreover, the mothers from our sample are could be iron depleted. Put together our results show that both maternal anaemia at delivery and newborn anaemia at birth are associated at different level, with haemoglobin progression in children during the first 18 months of age. These two risk factors could interact with each other and furthermore interact with feeding practices and have to be taken into account to define preventive strategy.

In this study, placental malaria was associated with a low haemoglobin level in the first18 months of life. This association was not significant during our first analysis [11]. This association has already been described by Redd *et al*. (1994), who have shown that placental malaria was associated with anaemia around 2 months of age [37]. In cases of iron deficiency during pregnancy, expression of placental transport proteins for iron increases, allowing a greater transport of iron to the foetus [38], [39]. In case of placental malaria, a thickening of the trophoblastic basement membrane has been described, damaging the placenta’s active transfer capacity [40]. It can therefore be assumed that placental malaria reduces the transfer of iron from the mother to her children, increasing the newborn’s iron deficiency. However, a more indirect explanation can be proposed that is consistent with the effect of placental malaria on haemoglobin level over time. Indeed, some authors have hypothesized that placental malaria is associated with an immune tolerance phenomenon [10], [41]. Children born of infected placenta are more susceptible to malaria infection. As both the number of malaria attacks during the follow-up and placental malaria were independently significantly associated with the level of haemoglobin in the first18 months, we can argue that children born of mothers with placental malaria are more susceptible not only to malaria but also to other infections, as we have recently shown in this same cohort [42]. These children, frailer and more often infected, have a higher risk of developing anaemia.

Our study also described a negative effect of sickle cell trait among trajectories. As this result was obtained during multivariate analysis with both variables (i.e. malaria and sickle cell) we can argue that the sickle cell trait effect is independent of the protection against clinical malaria classically described [43], [44]. Our hypothesis is that this effect could be explained by an intrinsic role of sickle cell trait in anaemia, even for heterozygous individuals. To our knowledge, one study has described an association between anaemia and sickle cell trait [45] but this association has not been confirmed to date. In addition, it has been described that haematuria, both microscopic and macroscopic, is one of the most frequent complication of sickle cell trait [46]–[48]. In our sample no macroscopic haematuria was found and we did not search a microscopic haematuria. However although we could hypothesize that children with sickle cell trait experienced microscopic haematuria, we cannot explain clearly the different effect of this variable in each group.

According with our protocol the children found anaemic were treated as proposed by the recommendation of the Ministry of Public Health of the Republic of Benin. They received haematinics which was prescribed by the nurses working in the public dispensaries of the area. The team involved in the follow-up was different and the population was monitored equivalently and independently of the Hb levels or of they life conditions. Furthermore, as the families involved in this program were very similar we do not think that a “cohort effect” could represent a limitation of the study.

In conclusion, this study, using latent class analysis models, shows that the occurrence of a malaria attack during follow-up, male gender, sickle cell trait carriage, children living in a polygamous family, children with good feeding practices, newborn anaemia, placental malaria were associated with haemoglobin level in children and that maternal anaemia was a predictor of a low haemoglobin level trajectory in children in their first 18 months of life. Latent class approach could be applied more frequently to analyse longitudinal data when the existence of groups with distinct pattern of evolution is suspected. This assumption cannot be adequately explored with mixed models. The prevalence of anaemia during pregnancy and in the newborn is very high in developing countries, 40% and 61%, respectively, in our study in Benin. There is a need to increase actions that target the prevention of maternal anaemia as well as placental malaria and newborn anaemia.
